# Adult Bovine-Derived Small and Large Intestinal Organoids: *In Vitro* Development and Maintenance

**DOI:** 10.1155/2023/3095002

**Published:** 2023-11-27

**Authors:** Minae Kawasaki, Gerald D. Dykstra, Craig S. McConnel, Claire R. Burbick, Yoko M. Ambrosini

**Affiliations:** ^1^Department of Veterinary Clinical Sciences, College of Veterinary Medicine, Washington State University, Pullman, Washington, USA; ^2^Department of Veterinary Microbiology and Pathology, College of Veterinary Medicine, Washington State University, Pullman, Washington, USA

## Abstract

Recent progress in bovine intestinal organoid research has expanded opportunities for creating improved *in vitro* models to study intestinal physiology and pathology. However, the establishment of a culture condition capable of generating organoids from all segments of the cattle intestine has remained elusive. Although previous research has described the development of bovine jejunal, ileal, and colonic organoids, this study marks the first report of successful bovine duodenal and rectal organoid development. Maintenance of these organoids through serial passages and cryopreservation was achieved, with higher success rates observed in large intestinal organoids compared to their small intestinal counterparts. A novel approach involving the use of biopsy forceps during initial tissue sampling streamlined the subsequent tissue processing, simplifying the procedure compared to previously established protocols in cattle. In addition, our study introduced a more cost-effective culture medium based on advanced DMEM/F12, diverging from frequently used commercially available organoid culture media. This enhancement improves the accessibility to organoid technology by reducing culture costs. Crucially, the derived organoids from the jejunum, ileum, colon, and rectum faithfully preserved the structural, cellular, and genetic characteristics of the *in vivo* intestinal tissue. This research underscores the significant potential of adult bovine intestinal organoids as a physiologically and morphologically relevant *in vitro* model. Such organoids provide a renewable and sustainable resource for a broad spectrum of studies, encompassing investigations into normal intestinal physiology in cattle and the intricate host-pathogen interactions of clinically and economically significant enteric pathogens.

## 1. Introduction

The field of intestinal biology research has seen a transformative shift with the adoption of three-dimensional (3D) cultures of intestinal epithelial cells [1]. These cultures, known as intestinal organoids, are derived from primary intestinal stem cells and faithfully replicate the structural complexity of the intestine. Comprising various cell lineages found in the intestinal epithelium, such as enterocytes, goblet cells, Paneth cells, and enteroendocrine cells, these organoids offer a unique opportunity to investigate intestinal physiology, nutrient absorption, and host-pathogen interactions [2, 3].

Notably, the application of bovine intestinal organoids as an *in vitro* model for infectious diseases has demonstrated their immense potential [4–7]. This is especially relevant in the context of the United States cattle industry, a significant contributor to the country's economy [8]. The health of cattle and their gut function directly impacts productivity, animal welfare, and production costs [9, 10]. In addition, cattle play a role in greenhouse gas emissions, particularly due to enteric methane, making the study of their gut health socially and economically significant [11].

Furthermore, cattle serve as a reservoir for pathogens with implications for human health [12]. Understanding host-pathogen interactions for these zoonotic enteric pathogens requires consideration of the specific gut segments they inhabit [13, 14]. Although intestinal organoid technology has been described for certain segments of the bovine gastrointestinal tract (i.e., the jejunum [5, 15], ileum [16, 17] and colon [18]), there remains a gap in our understanding of the duodenum and rectum. Addressing this gap is crucial for advancing our knowledge of pathophysiology and colonization processes.

To bridge these knowledge gaps and enhance our understanding of bovine gut physiology and pathogen interactions, we present a technique for culturing bovine intestinal organoids from five different segments of the adult bovine gut. This method, distinct from previous full-thickness tissue sampling approaches [4, 5, 7, 15–18], involves mucosa extraction using biopsy forceps and subsequent organoid cultivation with an in-house organoid growth media supplemented with growth factors and inhibitors. The resulting organoids are assessed for their fidelity to *in vivo* bovine gut traits through immunofluorescence and RT-qPCR analysis.

## 2. Materials and Methods

### 2.1. Animals and the Intestinal Tissue

**Figure 1:**
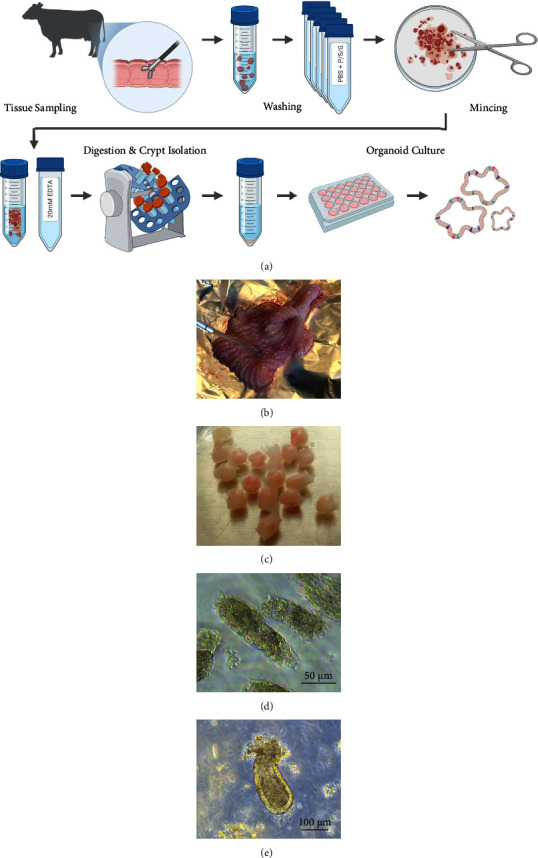
Development of bovine intestinal organoids. (a) A schematic demonstrating the flow of the technique used to generate organoids from the bovine intestine created with https://www.biorender.com/. (b–e) Gross and microscopic images showing tissue sampling (b), collected tissue samples after washing with the wash medium (c), isolated crypts (d), and a developing organoid 24 hours after seeding in Matrigel (e).

Intestinal tissue samples were collected from five approximately 15- to 18-month-old cattle at a local slaughterhouse. Signalments of donors are summarized in Supplementary Table 1. An overview of tissue sampling and subsequent processing techniques is illustrated in Figure 1(a). Five segments of the intestine, namely, the duodenum, jejunum, ileum, colon, and rectum, were identified and incised longitudinally to expose the mucosal surface (Figure 1(b)). Approximately, 10–15 pieces of the fresh tissue were sampled from each segment of the intestine using biopsy forceps (Hildyard Post-Nasal Biopsy Forceps, Med-Plus) (Figure 1(c)) and immediately placed in a wash medium consisting of ice-cooled Dulbecco's phosphate-buffered saline (PBS) (Gibco) supplemented with 1x penicillin/streptomycin (Gibco) and 25 *μ*g/mL gentamicin (Gibco). The samples were kept on ice until further processing at the laboratory.

### 2.2. Crypt Isolation and Organoid Generation

The samples were processed as described previously with some modifications to isolate intestinal crypts and obtain intestinal stem cells for organoid generation [19–21]. In brief, the pieces of the tissue were first washed with a sterile wash medium approximately 5 times or until the supernatant became clear by shaking vigorously and replacing the wash medium repeatedly. Once excess mucus and residual luminal contents were removed, the samples were resuspended into 20 mM EDTA solution and minced to small fragments in a standard tissue culture dish (Fisher Scientific) using sharp-pointed dissecting scissors (Fisher Scientific). The fragmented tissue was then collected into a 15 mL conical tube and incubated for 15 (jejunum and ileum), 30 (colon), or 60 (duodenum and rectum) minutes at 10 rpm at 4°C on a tube rotator (Fisher Scientific) to isolate intestinal crypts (Figure 1(d)). Following incubation, tissue fragments were allowed to settle to the bottom of the tube by gravity and the crypt-containing supernatant was collected in a new 15 mL conical tube. The crypts were pelleted by centrifugation of the supernatant at 200 × *g* at 4°C for 5 minutes. The supernatant was discarded, and the pellet was resuspended in Matrigel (Corning). The crypts were then seeded onto a 24-well plate in 30 *μ*L per well.

Following the polymerization of Matrigel at 37°C for 10–15 minutes, each well received 500 *μ*L of the organoid expansion medium (OEM) consisting of the components outlined in Table 1. The noggin and R-spondin-conditioned media were obtained by cultivating HEK293 cells engineered to secrete noggin (Baylor's College of Medicine) [22] and Cultrex HA-R-spondin1-Fc 293T cells (R&D Systems) as described previously [23]. 10 *μ*M rho-associated kinase inhibitor (ROCKi) Y-27632 (STEMCELL Technologies) and 100 nM glycogen synthase kinase (GSK) 3*β* inhibitor CHIR99021 (Sigma-Aldrich) were added to the OEM for the first 2–4 days of the culture and removed thereafter once organoids started growing in Matrigel (Figure 1(e)). The plate was incubated at 37°C with 5% CO_2_ and the OEM was renewed every other day for growth and maintenance of organoids. The growth of organoids was monitored daily, and images of organoids were taken using phase-contrast microscopy (DMi1, Leica).

### 2.3. Organoid Subculture and Maintenance

Organoids were serially passaged and expanded every 6–8 days as they grew to form lumina and villi-like budding structures following initial culture. For passaging, Matrigel-containing organoids were first dissociated by replacing the OEM with cold cell recovery solution (Corning) and incubating for 30 minutes at 4°C. Organoids were recovered from Matrigel by centrifuging at 200 × *g* at 4°C for 5 minutes and discarding the supernatant. Organoids were then disrupted by adding TrypLE Express (Gibco) and incubating in a 37°C water bath for 1 minute. Enzymatic disruption was stopped by adding the basal medium (advanced DMEM/F12 with GlutaMAX, HEPES, and penicillin/streptomycin). The dissociated organoids were pelleted down by centrifuging at 200 × *g* at 4°C for 5 minutes. The pellet was resuspended in Matrigel at an expansion ratio of approximately 1–8 wells and cultured as described above in 24- or 48-well plates with 500 *μ*L or 300 *μ*L of the culture medium, respectively.

### 2.4. Organoid Cryopreservation and Resuscitation

For cryopreservation, organoids were recovered from Matrigel as described above and washed once with the basal medium. Intact organoids were resuspended into the freezing medium consisting of 10% dimethyl sulfoxide (DMSO) in 90% fetal bovine serum (FBS) as described previously [20] and 0.5 mL of the suspension was immediately transferred to 1 mL cryovials. For resuscitation, cryovials were quickly thawed in a 37°C water bath and placed on ice. Organoids were washed once with the basal medium and cultured as described above.

### 2.5. Immunocytochemistry

Organoids were fixed with 4% paraformaldehyde (Thermo Scientific) for 15 minutes at room temperature, followed by permeabilization and blocking with 0.3% Triton X-100 (Thermo Scientific) for 15 minutes and 2% bovine serum albumin (BSA) (Cytiva) for 60 minutes. Primary antibodies and fluorescent probes against E-cadherin (1 : 200, BD Biosciences), EpCAM (1 : 200, Abcam), SOX9 (1 : 250, Abcam), and *Sambucus nigra* agglutinin (SNA) (1 : 100, Vector Laboratories) were diluted in 2% BSA (Supplementary Table 2) and incubated with the organoids overnight in darkness at 4°C. Organoids were then treated with a secondary antibody (Alexa Fluor 555-conjugated goat anti-rabbit IgG H&L, 1 : 1000, Abcam) and incubated for 1 hour at room temperature following washing with PBS.

Nuclei and F-actin were stained with 4′,6-diamidino-2-phenylindole dihydrochloride (DAPI) (1 : 1000, Thermo Scientific) and Alexa Fluor 647-conjugated phalloidin (1 : 400, Invitrogen), respectively, according to the manufacturers' recommendation (Supplementary Table 2). EdU assay (Invitrogen) was also performed following the manufacturer's protocol to detect actively proliferating cells within organoids. Organoids were washed with PBS and mounted on glass bottom dishes (Matsunami) using the ProLong Gold Antifade reagent (Invitrogen). Fluorescence imaging was performed using a white-light point scanning confocal microscope (SP8-X, Leica) and images were processed using LAS X (Leica). Immunocytochemistry was performed in at least two technical replicates using organoids derived from four independent donors.

For quantitative analysis, the number of cells that stained positive for SOX9, EdU, and SNA was counted on randomly selected representative images. The percent positive rate for each cell type was calculated by normalizing the positive cell number by the total number of nuclei. Three to ten independent fields of view from four biological replicates were assessed.

### 2.6. Gene Expression Analysis

Expression of genes indicative of specific intestinal cell types, namely, *LGR5* (stem cells), *ChrA* (enteroendocrine cells), *LyzC* (Paneth cells), *Muc2* (goblet cells), and *FABP2* (intestinal epithelial cells) [7, 24, 25], was determined and compared between organoids cultured in the OEM for 8 days and those treated with the organoid differentiation medium (ODM) for 4 days following initial culture in OEM for 4 days. The ODM was prepared by withdrawing Wnt3a, SB202190, and nicotinamide from OEM [1]. Total RNA was extracted from the jejunal, ileal, colonic, and rectal organoids in the OEM and ODM using the RNeasy Plus Mini Kit (Qiagen) and concentrations were quantified using NanoDrop One (Thermo Scientific). cDNA was synthesized using High-Capacity cDNA Reverse Transcription Kits (Applied Biosystems) and was subsequently used to carry out RT-qPCR reactions using PowerUp SYBR Green Master Mix (Applied Biosystems). Primers used in this study are listed in Supplementary Table 3. The primer sequence was amplified at 60°C for 40 cycles and generation of single product was confirmed using dissociation curves. Relative gene expression was calculated by applying the standard curve method and using *GAPDH*, *RPL0*, and *ACTB* as internal control [7, 26, 27]. RT-qPCR reactions were carried out in triplicate from one (jejunum) or three (ileum, colon, and rectum) biological replicates.

### 2.7. Statistical Analyses

Quantitative data were analyzed using R v.3.4.1 (the R foundation) and plotted using GraphPad Prism 9.5.1 (GraphPad Software). For immunocytochemistry, the percent positive rates were compared across the four intestinal segments using the Kruskal–Wallis test with a Bonferroni post-hoc test. For RT-qPCR analyses, data were compared between control (OEM-grown organoids) and ODM-treated organoids using Wilcoxon's signed rank test for independent samples. Results were presented as the mean ± standard error of the mean (SEM), with *p* values ≤0.05 being considered statistically significant.

## 3. Results

### 3.1. Establishment of Bovine Intestinal Organoids

**Figure 2:**
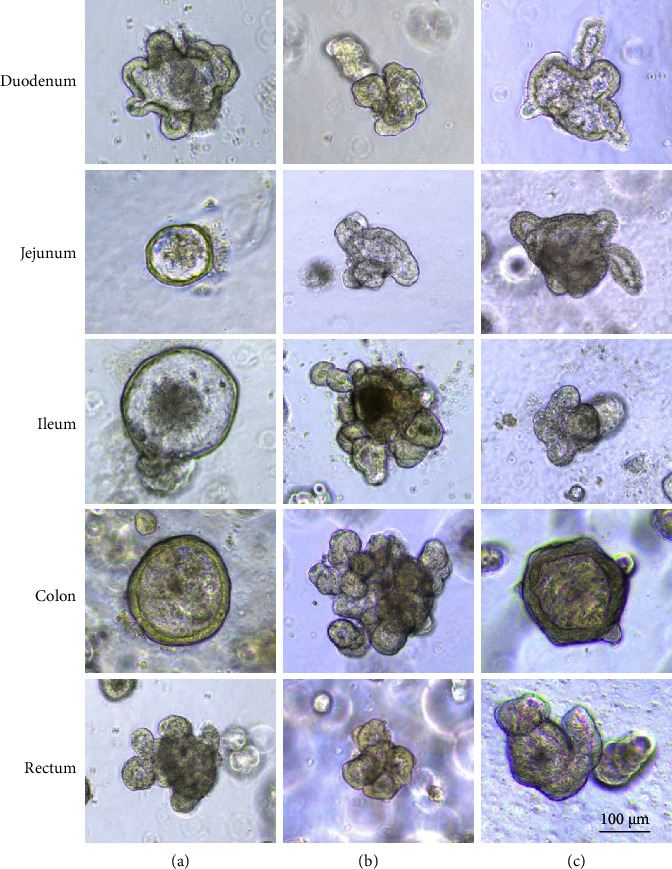
Representative images of bovine intestinal organoids taken with phase-contrast brightfield microscopy. (a) Organoids were generated from the freshly sampled tissue of the duodenum, jejunum, ileum, colon, and rectum of adult cattle. (b) Organoids from all the five segments were maintained over serial passages, with the highest passage numbers of P6 in the duodenum and >P10 in the jejunum, ileum, colon, and rectum. (c) Organoids from all the five intestinal segments were successfully resuscitated after cryopreservation for over 8 months. Images were captured between Day 2 and 12 either at P1-3 (a), P11 except for the duodenum which was imaged at P6 (b), or P4-5 (c), respectively.

*In vitro* 3D intestinal organoids were successfully generated from fresh tissue samples obtained via the biopsy technique from the duodenum, jejunum, ileum, colon, and rectum of five adult cattle (Figure 2(a)). Organoids generally formed spherical or budding luminal structures consistent with previous reports [5, 15–18, 28]. The success rate of initial organoid development using this biopsy technique was 100% in all the five segments (Table 2).

Serial *in vitro* passaging of these organoids demonstrated that organoids from the large intestine continuously and consistently grew and expanded for long term (>10 passages), yet those from the small intestine inconsistently retained their proliferating capability during early passages. Organoids which eventually lost their proliferating capacity were noted to disintegrate or cease to grow during early passages (i.e., passage 2–6; Supplementary Figure 1). These organoids were unable to expand in number or maintain culture thereafter; thus, we were unable to perform subsequent evaluations using immunocytochemistry and RT-qPCR.

Overall survival rates of organoids to passage 10 were 0% in the duodenum, 20% in the jejunum, 60% in the ileum, and 100% in both the colon and rectum (Table 2). Representative images of organoids following serial passages are shown in Figure 2(b). When organoids were cryopreserved in 10% DMSO in the FBS freezing medium and thawed after 8 months of storage, 100% of the organoid lines revived in all but duodenal organoids, where the resuscitation rate was 40% (Table 2, Figure 2(c)).

### 3.2. Characterization of Bovine Intestinal Organoids

**Figure 3:**
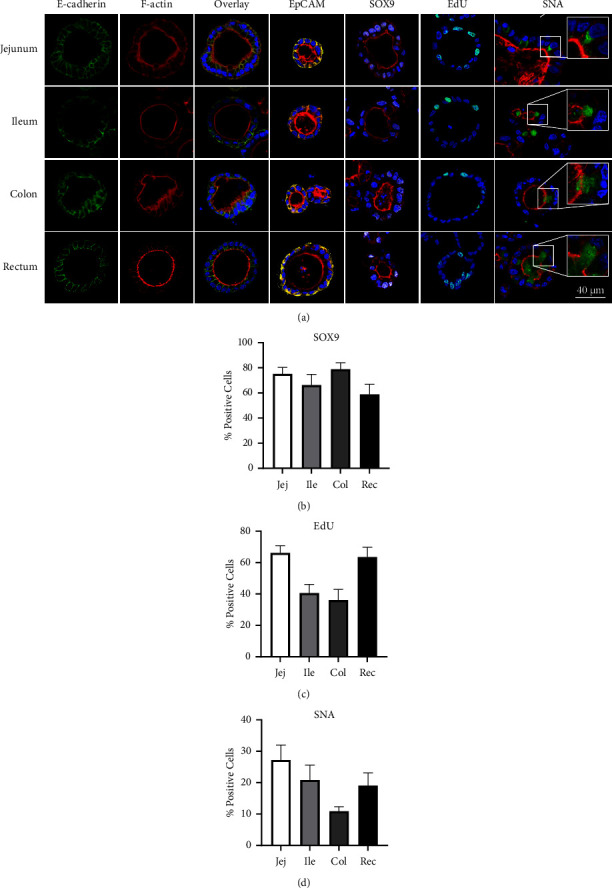
Immunocytochemical characterization of bovine intestinal organoids. (a) Confocal microscopy images of bovine intestinal organoids demonstrated the formation of basolateral epithelial adherens junctions (E-cadherin, green) and cellular polarity characterized with apical brush border (F-actin, red) and basal nuclei (DAPI, blue) and the presence of mixed cell populations including epithelial cells (EpCAM, yellow), stem cells (SOX9, yellow), actively proliferating cells (EdU, cyan), and mucin-producing goblet cells (SNA, green). (b–d) The bar graphs show quantification of the cells that stained positive for SOX9, EdU, and SNA normalized by the total numbers of nuclei. Three to ten independent fields of view from two technical and four biological replicates were assessed. The results are presented as the mean ± standard error of the mean (SEM).

Immunofluorescence staining of organoids demonstrated their basal-out structural characteristics with cellular polarization, heterogeneity of epithelial lineages, and retention of stemness and proliferative capacity in the jejunum, ileum, colon, and rectum (Figure 3(a)). More specifically, staining against E-cadherin confirmed the formation of basolateral epithelial adherence junctions. F-actin and nuclei stains confirmed cellular polarity characterized with apical brush border and basal nuclei, each of which was observed on luminal surfaces and towards the periphery of organoids, indicating basal-out cellular organization in organoids.

The epithelial nature of the organoid cells was confirmed by positive staining for EpCAM. The presence of SOX9-positive cells within organoids, as noted with distinct nuclei staining, indicated retention of stem cell characteristics which typify *in vivo* intestinal crypts. The EdU assay confirmed the maintenance of organoid proliferative capacity by depicting actively proliferating cells. In addition, fluorescein-labeled SNA, a sialic acid-specific lectin, was used to detect mucin which is produced by goblet cells. SNA-positive cells were noted with distinct cytoplasmic staining, where mucin is normally stored *in vivo*. Some SNA-positive cells appeared with mucus excreting into the organoid lumen at the apical surface of those cells, adding support to goblet cell differentiation. The mean percentages of positively stained cells for SOX9, EdU, and SNA across the four intestinal segments were 67.8 ± 22.3% (range: 58.8–78.8), 49.5 ± 20.9% (36.1–66.2), and 19.6 ± 12.1% (10.9–27.2), respectively (Figures 3(b)–3(d)). No significant difference was noted for all markers between the segments.

### 3.3. Relative Gene Expression of OEM-Grown vs. ODM-Treated Organoids

**Figure 4:**
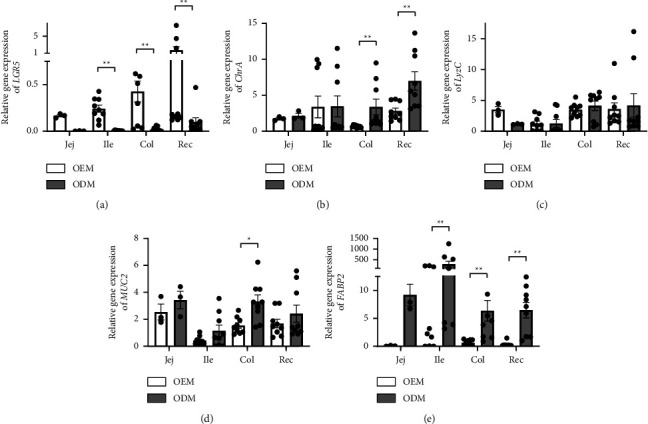
Relative gene expression of bovine intestinal organoids cultured in the expansion (OEM) and differentiation (ODM) media. RT-qPCR was performed to determine the expression of stem cell (*LGR5*, (a)), enteroendocrine cell (*ChrA*, (b)), Paneth cell (*LyzC*, (c)), goblet cell (*Muc2*, (d)), and intestinal epithelial cell (*FABP2*, (e)) marker genes using organoids cultured either in the OEM for 8 days or in the OEM for 4 days followed by the ODM for 4 days. The results are from samples derived from one (jejunum) or three (ileum, colon, and rectum) independent animals, with three technical replicates. The gene expression levels of each of the target genes were calculated relative to that of *GAPDH*, *RPL0*, and A*CTB* as internal control. The results are presented as the mean ± standard error of the mean (SEM). Statistical analysis was performed with Wilcoxon's signed rank test for independent samples. ^*∗*^*p* < 0.05, ^*∗∗*^*p* < 0.01.

RT-qPCR of organoids cultured in the OEM and ODM revealed variable expressions of genes indicative of specific cell types found in the intestine *in vivo*. The stem cell marker *LGR5* gene was expressed at significantly higher levels in ileal, colonic, and rectal organoids grown in the OEM compared with those treated with the ODM (*p* < 0.01; Figure 4(a)). The expression of *LGR5* was suppressed in ODM-treated jejunal organoids compared to those in OEM-grown organoids, although a difference was not noted between the groups (*p* = 0.25). In fact, *LGR5* expression was suppressed to barely detectable levels in ODM-treated jejunal, ileal, and colonic organoids. The expression of enteroendocrine cell marker *ChrA* gene was upregulated in ODM-treated colonic and rectal organoids (*p* < 0.01), whereas no difference was observed in jejunal and ileal organoids (*p* = 0.75 and 0.65, respectively; Figure 4(b)). The Paneth cell marker *LyzC* gene was expressed at higher levels in OEM-grown jejunal organoids (*p* = 0.25; Figure 4(c)). *LyzC* expression was slightly increased in ODM-treated organoids in the ileum, colon, and rectum, although no difference between the groups was noted (*p* = 0.50, 0.50, and 0.20, respectively). ODM treatment upregulated the expression of goblet cell marker *Muc2* and enterocyte marker *FABP2* genes in organoids of all the four segments (Figures 4(d) and 4(e)). Significant upregulation was noted in colonic organoids for *Muc2* (*p* = 0.02) and in the ileal, colonic, and rectal organoids for *FABP2* (*p* = 0.004 for all).

## 4. Discussion

The present study demonstrates that bovine intestinal organoids can be generated at a high success rate (100%) from the tissue obtained from the duodenum, jejunum, ileum, colon, and rectum using the biopsy technique previously described in dogs with some modifications [19–21]. Although the development of bovine jejunal, ileal, and colonic organoids has been described previously [5, 15–18], this is the first study which reports the successful development of bovine duodenal and rectal organoids. In addition, the use of biopsy forceps during initial tissue sampling is novel in cattle and was able to simplify subsequent tissue processing compared to the protocols previously described in cattle. Furthermore, the present study established a more cost-effective culture medium based on advanced DMEM/F12, which was similar but not identical to the media described to grow porcine jejunal and ileal organoids previously [29, 30]. This is in contrast to often employed commercially available organoid culture media used in previous studies, thus improving accessibility to the organoid technology through reduced culture costs.

In the present study, multiple small pieces of the partial thickness intestinal tissue were sampled using biopsy forceps. This contrasts with previously described techniques which usually sampled a portion of the full-thickness intestinal tissue in the field for transportation back to the laboratory for washing and further tissue processing [15–17]. Advantages of using biopsy forceps include the fact that samples can be collected with minimal debris or cleansed easily on site through wash media replacements in the case that large amount of ingesta or debris is noted at the time of sampling. Collecting cleaner samples is expected to minimize possible cellular damage resulting from bacterial exposure, potentially preserving cellular viability and improving the organoid generation rate. Although the importance of the minimizing gross contamination of intestinal specimens at the time of sampling has not been emphasized in previous studies, possible organoid development failure due to autolysis during transportation was reported in the gall bladder [31]. Therefore, it is likely a good practice to adopt cleaner sampling techniques when a delay in sample processing is expected (e.g., transporting to a distant laboratory or sampling multiple animals consecutively). Moreover, the present sampling technique was simpler as it did not require scraping off the mucosal layer and villi using a glass slide before mincing samples into small fragments [15–17]. No difference in crypt isolation and subsequent organoid generation efficiencies was noted between the samples collected using biopsy forceps before or after scraping with a glass slide (data not shown). Potential disadvantages of using biopsy forceps might include a reduction in the amount of the tissue that is available for further processing as compared to previous techniques; hence, the number of crypts isolated from the samples also would be reduced. However, the current study demonstrated that sampling of 10–15 pieces of biopsy tissues from each segment of the intestine resulted in the isolation of a sufficient number of crypts to generate organoids successfully and consistently during the initial cultivation.

Organoids derived from all the five segments of the intestine were successfully resuscitated following cryopreservation and all, but duodenal organoids were maintained and expanded stably for a long term through serial passages without losing their proliferating capacity. The present study observed the maintenance of stable culture in all but duodenal organoids for greater than 15 passages at the time of manuscript preparation, which was similar to or greater than previous observations [5, 15, 16]. Cryopreservation and resuscitation of viable bovine intestinal organoids was only reported previously with jejunal and ileal organoids [4, 5, 7, 15–17]. The commercially available cryopreservation medium has been used most commonly, with two studies preparing their own freezing medium consisting of 10–20% DMSO in the culture medium. The freezing medium used in the present study was adopted from the work reported with canine intestinal organoids and was not tested previously in bovine organoids [20]. Therefore, the present results demonstrate that rectal organoids can be maintained for long term through serial passages similar to jejunal, ileal, and colonic organoids. Furthermore, our results demonstrate that duodenal, colonic, and rectal organoids as well as jejunal and ileal organoids can be cryopreserved for later use and the freezing medium previously reported in canine intestinal organoids is applicable to bovine intestinal organoids.

Success rates for cryopreservation and long-term cultivation varied across the organoids derived from different bovine intestinal segments. High success rates were generally observed in colonic and rectal organoids, suggesting that our OEM composition provide a more favorable *in vitro* environment for the growth of large intestinal organoids. In contrast, duodenal organoids from all the five donors lost proliferating capacity following several passages and failed to expand to provide enough organoids for subsequent characterization with immunocytochemistry and RT-qPCR. Although exact reasons for this failure remain unknown, it is possible that bovine duodenal organoids require additional support for long-term survival. For example, this might take the form of continuous ROCKi supplementation to the OEM as was the case in rabbits in contrast to other species [32] or the addition of prostaglandin E2 to a culture medium as was described in the human duodenal organoid culture [33]. These treatments were helpful in reducing dissociation-associated apoptosis in human embryonic stem cell cultures [34, 35] and in promoting survival and growth of chicken embryo intestinal organoids [36]. Further work is necessary to optimize culture conditions in order to achieve long-term cultivation and expansion of bovine small intestinal organoids, especially duodenal organoids. However, the ability to maintain organoids *in vitro* and revitalize them at a later time through cryopreservation has important implications for livestock research. For instance, researchers can establish a Biobank of valuable samples, share resources among institutions regardless of their geographical distance, expand organoids for use in multiple independent research projects, or apply their use to various investigations simultaneously, all while limiting the number of animals required for different studies.

Characterization of the jejunal, ileal, colonic, and rectal organoids by means of immunocytochemistry confirmed the presence of a multilineage cellular population including intestinal epithelial stem cells, intestinal epithelial cells, and goblet cells. It also confirmed the presence of cellular polarity, adherens junction formations, and cellular proliferative capacity as previously reported both *in vivo* and *in vitro* of bovine and other species [5, 17, 18]. Furthermore, RT-qPCR of jejunal, ileal, colonic, and rectal organoids demonstrated expression of genes which are specific to differentiated cell types such as enterocytes (*FABP2*), enteroendocrine cells (*ChrA*), Paneth cells (*LyzC*), and goblet cells (*Muc2*), as well as a the stem cell marker gene (*LGR5*) in all the four segments. These results suggest that the bovine intestinal organoids generated in this study can serve as an *in vitro* bovine intestinal model as they retained the regenerative capacity of crypts and possessed structural and physiological similarity to the *in vivo* intestinal tissue.

The percentages of cells retaining stemness, displaying active proliferative capacity, and differentiating into mucus-producing goblet cells were found to be comparable to or even higher than previously reported observations in both the human and porcine *in vivo* intestinal as well as *in vitro* intestinal systems [37, 38]. Moreover, the distribution of stem cells and actively proliferating cells across the four segments aligned with the patterns observed in the *in vivo* intestine, where crypt niches and mitotic rates of intestinal stem cells are consistent between the small and large intestines [39]. In contrast, the relative abundance of mucus-secreting goblet cells between the small and large intestines, as observed in our study, deviated from the typical *in vivo* trend, where the number of goblet cells generally increases from the proximal to the distal intestine [40, 41]. Interestingly, a similar trend to our findings has been documented in a study involving human small and large intestinal organoids [42]. While further investigations are warranted to elucidate the precise reasons behind this discrepancy between *in vivo* and *in vitro* models, their observation of low ATOH1 expression in large intestinal organoids suggests the potential existence of an alternative pathway, distinct from the well-known Wnt and Notch pathways that regulates goblet cell differentiation in the large intestine.

Expression of *LGR5* and *LyzC* genes decreased or remained unchanged when organoids were treated with the ODM as compared to being OEM-grown. These results indicated that our ODM does not support maintenance of stem cells nor differentiation of stem cells into Paneth cells, which is somewhat expected as Wnt signaling plays a pivotal role in these functions [1, 43]. On the other hand, treatment with the ODM promoted the expression of *FABP2*, *ChrA*, and *Muc2* genes, indicating that ODM-treated organoids are a superior model of the *in vivo* intestine than OEM-grown organoids due to the exhibition of a greater heterogeneity of cellular populations related to intestinal epithelial cell lineages.

As a limitation, the present study did not evaluate the direct gene expression of the donor tissue; thus, it is not possible to compare and discuss similarities or differences in genetic properties between the organoids and their originating tissues. However, previous studies reported an overlapping gene expression pattern between bovine intestinal organoids and the *in vivo* tissue [15, 16, 44]. This characteristic has also been reported for other types of organoids such as the human and murine liver [45, 46]. The highest level of *FABP2* expression was in ileal organoids followed by jejunal and large intestinal organoids in this study, which is in accordance with a previous report that evaluated gene expression in various locations of intestinal tissues collected from Jersey calves [47]. Indistinguishable levels of *Muc2* gene expression across jejunal, ileal, colonic, and rectal organoids was also in agreement with a previous study using tissues collected from 4-year-old cows [48]. Furthermore, a high resemblance of organoids and the *in vivo* tissue as compared to a cell line was documented by applying a transcriptome analysis on the porcine jejunum and human duodenum [49, 50]. Our bovine intestinal organoids treated with the ODM offer an *in vitro* model that aligns more closely with physiological relevance compared to conventional cell culture methods or organoids grown in the OEM.

## 5. Conclusion

The present study demonstrates that bovine intestinal organoids can be generated from tissues of the duodenum, jejunum, ileum, colon, and rectum using a biopsy sample technique and OEM prepared in our laboratory, filling in a knowledge gap in bovine organoid technology. Maintenance of organoids through serial passages and cryopreservation was feasible in all five segments, with more consistent and higher success rates observed in large intestinal organoids as compared to small intestinal organoids. Organoids from the jejunum, ileum, colon, and rectum retained structural, cellular, and genetic resemblance to the *in vivo* intestinal tissue. These results suggest that adult bovine intestinal organoids offer a morphologically and physiologically relevant *in vitro* system that can be used as a long-term renewable resource for various studies including the investigation of normal intestinal physiology in cattle and host-pathogen interactions of clinically and economically important enteric pathogens with public health significance.

## Figures and Tables

**Table 1:** Composition of the organoid expansion medium (OEM) used to generate and maintain bovine intestinal organoids.

Reagents	Manufacturer	Final concentration
Advanced DMEM/F12	Gibco	NA
Noggin conditioned medium	Made in the laboratory	10%
R-spondin-conditioned medium	Made in the laboratory	20%
Recombinant murine Wnt-3a	PeproTech	100 ng/mL
A-83-01	Sigma-Aldrich	500 nM
B27	Gibco	1x
Murine EGF	R&D Systems	50 ng/mL
Gastrin	Sigma-Aldrich	10 nM
N2	R&D Systems	1x
Nicotinamide	Sigma-Aldrich	10 mM
N-Acetyl-L-cysteine	MP Biomedicals	1 mM
SB202190	Sigma-Aldrich	10 *μ*M
Primocin	InvivoGen	100 *μ*g/mL
Penicillin/streptomycin	Gibco	1x
GlutaMAX	Gibco	2 mM
HEPES	Gibco	10 mM

Manufacturers and final concentrations of each reagent are listed.

**Table 2:** Summary of development, maintenance, and cryopreservation of organoids.

Intestinal segments	Initial organoid development (P0)	Maintenance through serial passages		Resuscitation following cryopreservation
		Short-term (up to P5)	Long-term (>P10)	
Duodenum	5/5 (100%)	1/5 (20%)	0/5 (0%)	2/5 (40%)
Jejunum	5/5 (100%)	1/5 (20%)	1/5 (20%)	2/2 (100%)
Ileum	5/5 (100%)	4/5 (80%)	3/5 (60%)	4/4 (100%)
Colon	5/5 (100%)	5/5 (100%)	5/5 (100%)	5/5 (100%)
Rectum	5/5 (100%)	5/5 (100%)	5/5 (100%)	5/5 (100%)

Success rates for initial organoid development (P0) from the freshly sampled intestinal tissue, short- (up to P5) and long-term (>P10) maintenance of organoids through serial passages, and resuscitation following cryopreservation are listed.

## Data Availability

All the data relevant to the study are included in the article or uploaded as supplementary information.

## Supplementary Materials

Supplementary Materials Supplementary Table 1: summary of the signalment of animals sampled in this study. Animal ID, breed, sex, and estimated age in months are listed. Supplementary Table 2: primary antibodies and fluorescent probes used to characterize bovine intestinal organoids. Target, host species, dilution, and manufacturers of each antibody/probe are listed. Supplementary Table 3: primers used to evaluate the gene expression of bovine intestinal organoids. Gene name, types/markers, and forward and reverse sequences are listed. Supplementary Figure 1: representative images of disintegrating bovine intestinal organoids taken with phase-contrast brightfield microscopy. Duodenal, jejunal, and ileal organoids derived from some individuals started to disintegrate following routine passaging during early passages. These organoids eventually lost their proliferating capacity and were unable to maintain in long term through serial passages.
