# Supplementary material for: Adult Bovine-Derived Small and Large Intestinal Organoids: In Vitro Development and Maintenance
Source: J Tissue Eng Regen Med. 2023 Nov 27;2023:3095002. doi: 10.1155/2023/3095002 (PMC11175594; doi:10.1155/2023/3095002)
Supplement: Supplementary Materials — Supplementary Table 1: summary of the signalment of animals sampled in this study. Animal ID, breed, sex, and estimated age in months are listed. Supplementary Table 2: primary antibodies and fluorescent probes used to characterize bovine intestinal organoids. Target, host species, dilution, and manufacturers of each antibody/probe are listed. Supplementary Table 3: primers used to evaluate the gene expression of bovine intestinal organoids. Gene name, types/markers, and forward and reverse sequences are listed. Supplementary Figure 1: representative images of disintegrating bovine intestinal organoids taken with phase-contrast brightfield microscopy. Duodenal, jejunal, and ileal organoids derived from some individuals started to disintegrate following routine passaging during early passages. These organoids eventually lost their proliferating capacity and were unable to maintain in long term through serial passages. [file 3095002.f1.pdf]

Supplementary Materials

Supplementary Table 1: Summary of signalment of animals sampled in this study.

Animal ID	Breed	Sex	Estimated Age (Months)
1	Hereford	MN	18
2	Angus x Charolais	MN	18
3	Charolais	MN	18
4	Angus	MN	15-18
5	Angus	F	15-18

Animal ID, breed, sex and estimated age in months are listed.

Supplementary Table 2: Primary antibodies and fluorescent probes used to characterize bovine intestinal organoids.

Antibodies/ Fluorescent Probes	Target	Host Species	Dilution	Manufacturer (Catalog No.)
E-cadherin	Adherens junctions	Mouse	1:200	BD Biosciences (560061)
EpCAM	Epithelial cells	Rabbit	1:200	Abcam (ab71916)
SOX9	Stem cells	Rabbit	1:250	Abcam (ab185966)
SNA	Lectin biding mucin (Goblet cells)	-	1:100	Vector Laboratories (FL-1301)
DAPI	Nuclei	-	1:1000	Thermo Scientific (62248)
Phalloidin	Actin filaments	-	1:400	Invitrogen (A22287)
EdU	Actively proliferating cells	-	5 µM	Invitrogen (C10337)

Target, host species, dilution, and manufacturers of each antibody/probe are listed.

Supplementary Table 3: Primers used to evaluate gene expression of bovine intestinal organoids.

Gene Name	Gene Type/Marker	Forward	Reverse
Glyceraldehyde 3-phosphate dehydrogenase (*GAPDH*)	Internal Control	ATCTCGCTCCTGGAAGATG	TCGGAGTGAACGGATTCG
Ribosomal protein L0 (*RPL0*)	Internal Control	CAACCCTGAAGTGCTTGACAT	AGGCAGATGGATCAGCCA
β-actin (*ACTB*)	Internal Control	CTAGGCACCAGGGCGTAATG	CCACACGGAGCTCGTTGTAG
Leucine-rich repeat-containing protein-coupled receptor 5 (*LGR5*)	Stem cell	ACTTTCCAGCAGTTGTTCAGC	GAATAGACGACAGGCGGTTG
Chromogranin A (*ChrA*)	Enteroendocrine cell	GGGACACTGAGGTGATGAAG	GTCGCAGGATTGAGAGGAT
Lysozyme C (*LyzC*)	Paneth cell	TTCCTTTCTGTTGCTGTCCA	AGCCATCCAGTCCAAGTTTC
Mucin 2 (*MUC2*)	Goblet cell	TTCGACGGGAGGAAGTACAC	TTCACCGTCTGCTCATTCAG
Fatty acid binding protein 2 (*FABP2*)	Intestinal epithelial cell	GGAGGGAGATAAACTTGTCGG	ATCTGTGTTCTGGGCAATGC

Gene name, types/markers, and forward and reverse sequences are listed.

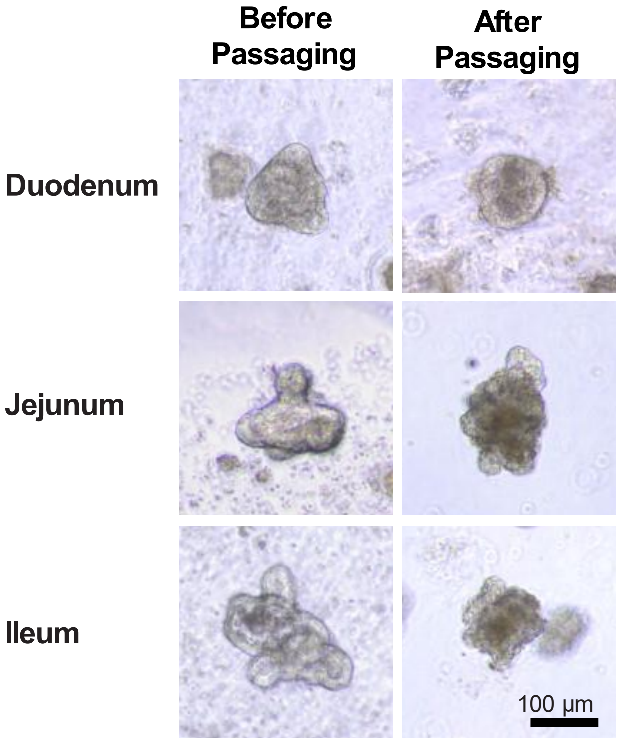

Supplementary Figure 1: Representative images of disintegrating bovine intestinal organoids taken with phase-contrast brightfield microscopy. Duodenal, jejunal and ileal organoids derived from some individuals started to disintegrate following routine passaging during early passages. These organoids eventually lost their proliferating capacity and were unable to maintain long-term through serial passages.
